# Fast-Acquiring High-Quality Prony Series Parameters of Asphalt Concrete through Viscoelastic Continuous Spectral Models

**DOI:** 10.3390/ma15030716

**Published:** 2022-01-18

**Authors:** Yan Zhang, Yiren Sun

**Affiliations:** 1 City Institute, Dalian University of Technology, Dalian 116600, China; yanzhang_dut@163.com; 2 School of Transportation and Logistics, Dalian University of Technology, Dalian 116024, China

**Keywords:** asphalt concrete, Prony series, Havriliak-Negami (HN) model, 2S2P1D model, continuous relaxation and retardation spectra

## Abstract

Prony series representations have been extensively applied to characterizing the time-domain linear viscoelastic (LVE) material functions for asphalt concrete. However, existing methods that can generate high-quality Prony series parameters (i.e., discrete spectra) mostly involve complicated programming algorithms, which poses a challenge for quick access of Prony series parameters. Also, very limited research has been devoted to establishing methods for simultaneously determining both retardation and relaxation spectra. To resolve these issues, this study presented a practical approach to fast acquiring high-quality Prony series parameters for both relaxation modulus and creep compliance of asphalt concrete by using the complex modulus test data. The approach adopts the analytical representations of the continuous relaxation and retardation spectra from the Havriliak-Negami (HN) and 2S2P1D complex modulus models to directly determine the discrete spectra, and the elastic constants, *E*_e_ and *D*_g_, for both LVE modulus and compliance functions are further calculated by fitting the corresponding generalized Maxwell model representations to smoothed data from the storage modulus representations of the HN and 2S2P1D complex modulus models. In this way, all the procedures in the proposed method can be easily implemented in Microsoft Excel. The results showed that the HN and 2S2P1D models yielded slightly different continuous spectral patterns at shorter relaxation times and longer retardation times. However, at the region covered by the test data, the continuous spectra of the two complex modulus models were very close to each other. Thus, the two models can generate comparable Prony series parameters within the time or frequency range covered by the test data. Considering that the quality of the resulting Prony series parameters are closely related to the master curve models used for presmoothing, the HN and 2S2P1D models were compared with the conventional Sigmoidal model. Additionally, the Black diagram was recommended for examining the quality of the complex modulus test data before constructing the master curves.

## 1. Introduction

Asphalt concrete, which has been paved on most roadways in the world, is a typical particulate composite with a viscoelastic matrix. In engineering applications, it is commonly regarded as a linear viscoelastic (LVE) material [1,2,3]. As such, many mechanical tests based on the LVE theory, like the static relaxation and creep tests and the dynamic complex modulus test, can be used for characterizing its LVE behavior. Theoretically, the properties from these tests such as the relaxation modulus, creep compliance and complex modulus are equivalent [4,5,6]; however, for a practical purpose, the uniaxial compressive complex modulus test has been widely accepted as a standard LVE material characterization test. After the complex modulus test data is obtained, it is usually required to extract the LVE information from the test data through mathematical models.

In the LVE theory, the generalized Maxwell model and generalized Kelvin model appear to be the most commonly used models for describing both time- and frequency-domain material functions, and they have been implemented into many commercial numerical simulation programs, e.g., ABAQUS, ANSYS and COMSOL Multiphysics [7,8,9,10,11]. This can be primarily attributed to their high computational efficiency and wide applicability [3,12,13]. The two models are composed of linear springs and dashpots linked in different configurations, and mathematically yield the so-called Prony series expressions for the relaxation modulus and creep compliance in time domain [3,12,14]. The Prony series expressions are not only very convenient to be converted analytically into the frequency-domain complex modulus and compliance, but also can considerably facilitate the computation of the convolution integrals for the LVE constitutive equations due to the presence of decaying exponential terms. Therefore, accurate and efficient identification of the Prony series parameters (i.e., discrete relaxation and retardation spectra) is crucial to the subsequent performance analysis and prediction of asphalt pavement or mixtures.

To date, researchers have proposed various methods for determining the Prony series parameters. Several representative approaches that apply directly to raw data in the time or frequency domain have been widely used for LVE materials, e.g., the collocation method by Schapery [15], the multidata method by Cost and Becker [16], and the windowing method by Tschoegl and Emri [17]. Nonetheless, these classic schemes would encounter difficulties when utilized for asphalt concrete. Two major issues, namely negative spectrum strengths and local spectrum oscillations, occur frequently due to the narrowband nature of the Prony series terms and significant scatters in test data. To address these problems, presmoothing techniques have been introduced by using broadband functions, like the Sigmoidal model [2], power-law series [3], Huet-Sayegh model [18], Havriliak-Negami (HN) model [14] and 2S2P1D model [19,20]. The use of these broadband functions not only improves the quality of the test data, but facilitates the data shift in accordance with the time-temperature superposition principle (TTSP) during the construction of master curves. In view of the equivalence of the LVE material functions, some interconversion algorithms [13,21,22] were also presented to calculate the Prony series parameters of the relaxation functions from the retardation functions, or vice versa.

On the other hand, the continuous spectrum-based methods attract increasing attention from the asphalt paving research community in that they are able to eliminate negative spectrum strengths and excessive parameters. Levenberg [23] developed a continuous relaxation spectrum model for asphalt concrete by using a lognormal distribution function. However, this model is symmetrical on the logarithmic timescale and thus may not be appropriate for all mixtures. Zhao et al. [2] established a confining pressure dependent continuous relaxation spectrum by considering the relationship between the relaxation spectrum and storage modulus. Luo et al. [24] and Lv et al. [25] respectively deduced continuous relaxation spectra from a modified power law-based relaxation modulus model and a generalized Sigmoidal model-based storage modulus model. Nevertheless, these works were all concentrated on the relaxation spectrum, and thus may be inconvenient for those who need fast solutions for both retardation and relaxation functions. Aiming at this issue, Sun et al. [26] presented a numerical approach to determining a continuous spectrum from the other. Bhattacharjee et al. [27] and Zhang et al. [28] calculated the two continuous spectra from storage modulus and storage compliance separately based on the Sigmoidal function and the generalized Sigmoidal function; however, due to the inconsistency of the model parameters of the storage modulus and storage compliance, the LVE relationship cannot be strictly satisfied.

Although there have been so many methods developed for determining the Prony series parameters as mentioned above, most of them involve complicated programming algorithms, which poses a challenge for quick access of Prony series parameters. Furthermore, very limited research has been devoted to establishing approaches for determining both retardation and relaxation spectra at the same time. To deal with these problems, this study gave a practical approach by adopting analytical representations of the continuous relaxation and retardation spectra from two complex-valued models, and all the procedures in the method can be easily implemented in Microsoft Excel.

## 2. Materials and Complex Modulus Test

**Figure 1:**
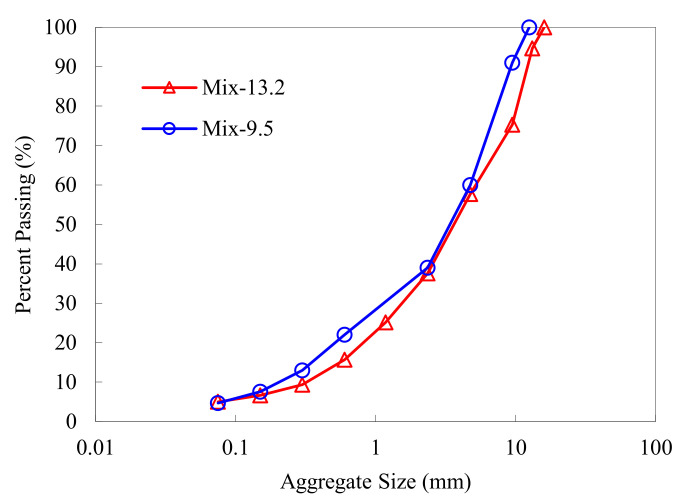
Aggregate gradations of Mix-13.2 and Mix-9.5.

Two dense-graded asphalt mixtures, denoted as Mix-13.2 and Mix-9.5 herein, were prepared for the complex modulus testing. Mix-13.2 had a nominal maximum aggregate size (NMAS) of 13.2 mm. The coarse aggregates and asphalt binder used were limestone and PG 58-22 unmodified asphalt, respectively. Mix-9.5 had a NMAS of 9.5 mm. The coarse aggregates and asphalt binder used were granite and PG 64-22 neat asphalt, respectively. The asphalt contents of Mix-13.2 and Mix-9.5 were 3.9% and 5.7%, respectively. Figure 1 presents the aggregate gradations of the two asphalt mixtures.

The complex modulus tests were performed on all specimens of the two mixtures in accordance with the standard testing method AASHTO T342 [29]. The two mixtures were first compacted using the Superpave Gyratory Compactor and then trimmed into the final cylindrical specimens (150 mm in height and 100 mm in diameter) containing an air void content of 4 ± 1%. For each mixture, three replicate specimens were fabricated.

The complex modulus testing was conducted on a universal testing machine (UTM). A stress-controlled compressive mode was employed for all the complex modulus tests. For Mix-13.2, five testing temperatures (−10, 5, 20, 35 and 50 °C) and seven loading frequencies (0.1, 0.5, 1, 5, 10, 20 and 25 Hz) were adopted, and for Mix-9.5, five testing temperatures (−16, 4, 24, 40 and 50 °C) and six loading frequencies (0.1, 0.5, 1, 5, 10 and 25 Hz) were adopted. During testing, the strain was kept between 50~150 με and the accumulated strain was controlled below 1500 με to ensure LVE measurements. By means of the obtained stress and strain data, two quantities, i.e., the dynamic modulus |E*| and phase angle *φ*, can be calculated as follows:

(1) $$\left|E^{*}\right|=\frac{\sigma_{0}}{\varepsilon_{0}}$$

(2) $$\varphi =\frac{\Delta t}{t_{p}}\times 360^{\circ }$$

where *σ*_0_ and *ε*_0_ are the amplitudes of the axial stress and strain; Δ*t* is the time lag of the strain curve behind the stress curve; *t*_p_ is the loading period. The dynamic modulus |E*|, which is the absolute value of the complex modulus E*, characterizes the resistance to deformation of a viscoelastic material, whereas the phase angle *φ* characterizes the extent to which the viscoelastic material behaves like a viscous liquid (*φ* = 90°) or an elastic solid (*φ* = 0°).

## 3. Methodology

### 3.1. Viscoelastic Master Curve Models

In this study, two models, i.e., the HN model and the 2S2P1D model, were employed to build the complex modulus master curves of the asphalt mixtures. The HN model has five parameters and is represented by [30]:

(3) $$E^{*}\left(\omega \right)=E_{g}+\frac{E_{e}-E_{g}}{{\left[1+{\left(i\omega \tau_{0}\right)}^{\alpha }\right]}^{\beta }}$$

where *E*_g_ is the glassy modulus; *E*_e_ is the equilibrium modulus; *ω* = 2π*f* is the angular frequency; *f* is the frequency; $i=\sqrt{-1}$; *α*, *β* and *τ*_0_ are model parameters and they respectively control the width, asymmetry and horizontal position of the relaxation spectrum.

The real part *E*′ and imaginary part *E*″ of the complex modulus E* have the following relationship with the dynamic modulus |E*| and phase angle *φ*:

(4) $$E^{*}=E^{\prime }+iE^{\prime \prime }=\left|E^{*}\right|\cos\varphi +i\left|E^{*}\right|\sin\varphi$$

where *E*′ is the storage modulus; *E*″ is the loss modulus. Thus, the dynamic modulus |E*| and phase angle *φ* can be calculated using the storage modulus *E*′ and the loss modulus *E*″, as follows

(5) $$\left|E^{*}\right|=\sqrt{{E^{\prime }}^{2}+{E^{\prime \prime }}^{2}}\;\mathrm{and}\;\varphi =\arctan\frac{E^{\prime \prime }}{E^{\prime }}$$

From the HN model, the representations of the storage modulus and loss modulus can be analytically separated out according to De Moivre’s formula, as the following [30]:

(6) $$E^{\prime }=E_{g}+\frac{\left(E_{e}-E_{g}\right)\cos\left(\beta \psi \right)}{{\left[1+2\omega^{\alpha }\tau_{0}^{\alpha }\cos\left(\alpha \pi /2\right)+\omega^{2\alpha }\tau_{0}^{2\alpha }\right]}^{\beta /2}}$$

(7) $$E^{\prime \prime }=\frac{\left(E_{g}-E_{e}\right)\sin\left(\beta \psi \right)}{{\left[1+2\omega^{\alpha }\tau_{0}^{\alpha }\cos\left(\alpha \pi /2\right)+\omega^{2\alpha }\tau_{0}^{2\alpha }\right]}^{\beta /2}}$$

(8) $$\psi =\arctan\frac{\omega^{\alpha }\tau_{0}^{\alpha }\sin\left(\alpha \pi /2\right)}{1+\omega^{\alpha }\tau_{0}^{\alpha }\cos\left(\alpha \pi /2\right)}$$

Obviously, with the analytical expressions of the storage and loss moduli, those for the dynamic modulus and phase angle are also available according to Equation (5).

The 2S2P1D model, composed of two spring elements, two parabolic elements and a dashpot element, possesses seven parameters and has the following mathematical form [19]:

(9) $$E^{*}\left(\omega \right)=E_{e}+\frac{E_{g}-E_{e}}{1+\alpha {\left(i\omega \tau_{0}\right)}^{-k}+{\left(i\omega \tau_{0}\right)}^{-h}+{\left(i\omega \beta \tau_{0}\right)}^{-1}}$$

where *α*, *k* and *h* (0 < *k* < *h* < 1) are the parameters of the two parabolic elements; *τ*_0_ is a temperature-dependent parameter; *β* is the parameter associated with the Newtonian viscosity of the dashpot element, *η* = (*E*_g_ − *E*_e_) *βτ*_0_.

According to De Moivre’s formula, the storage and loss moduli representations of the 2S2P1D model can also be derived, as follows:

(10) $$E^{\prime }=E_{e}+\frac{\left(E_{g}-E_{e}\right)\left(1+A\right)}{{\left(1+A\right)}^{2}+B^{2}}$$

(11) $$E^{\prime \prime }=\frac{\left(E_{e}-E_{g}\right)B}{{\left(1+A\right)}^{2}+B^{2}}$$

(12) $$A=\alpha {\left(\omega \tau_{0}\right)}^{-k}\cos\left(k\pi /2\right)+{\left(\omega \tau_{0}\right)}^{-h}\cos\left(h\pi /2\right)$$

(13) $$B=-\alpha {\left(\omega \tau_{0}\right)}^{-k}\sin\left(k\pi /2\right)-{\left(\omega \tau_{0}\right)}^{-h}\sin\left(h\pi /2\right)-{\left(\omega \beta \tau_{0}\right)}^{-1}$$

Since the storage and loss moduli of the HN and 2S2P1D models can all be derived analytically from the corresponding complex-valued models, they accurately meet the Kronig–Kramers relation that correlates the real and imaginary parts of the response to a harmonic load to each other theoretically [31].

Besides, in the viscoelastic theory, when the representation of the complex modulus E* is known, the complex compliance D* can be analytically obtained by taking the inverse of the complex modulus, as follows:

(14) $$D^{*}=D^{\prime }-iD^{\prime \prime }=1/E^{*}$$

(15) $$D^{\prime }=\frac{E^{\prime }}{{E^{\prime }}^{2}+{E^{\prime \prime }}^{2}}$$

(16) $$D^{\prime \prime }=\frac{E^{\prime \prime }}{{E^{\prime }}^{2}+{E^{\prime \prime }}^{2}}$$

where *D*′ is the storage compliance; *D*″ is the loss compliance.

### 3.2. Continuous Relaxation and Retardation Spectra

For a LVE material, the modulus functions in the time and frequency domains can be uniformly expressed using the continuous relaxation spectrum *H*(*ρ*) through integral forms [31]:

(17) $$E^{\prime }\left(\omega \right)=E_{e}+\int_{-\infty }^{\infty }H\left(\rho \right)\frac{\omega^{2}\rho^{2}}{1+\omega^{2}\rho^{2}}d\ln\rho$$

(18) $$E^{\prime \prime }\left(\omega \right)=\int_{-\infty }^{\infty }H\left(\rho \right)\frac{\omega \rho }{1+\omega^{2}\rho^{2}}d\ln\rho$$

(19) $$E\left(t\right)=E_{e}+\int_{-\infty }^{\infty }H\left(\rho \right)e^{-t/\rho }d\ln\rho$$

where *ρ* is the relaxation time; *E*(*t*) is the relaxation modulus; *t* is the loading time.

Similarly, the compliance functions of a LVE material in the time and frequency domains can be uniformly expressed using the continuous retardation spectrum *L*(*τ*) through integral forms [31]:

(20) $$D^{\prime }\left(\omega \right)=D_{g}+\int_{-\infty }^{\infty }L\left(\tau \right)\frac{1}{1+\omega^{2}\tau^{2}}d\ln\tau$$

(21) $$D^{\prime \prime }\left(\omega \right)=\int_{-\infty }^{\infty }L\left(\tau \right)\frac{\omega \tau }{1+\omega^{2}\tau^{2}}d\ln\tau$$

(22) $$D\left(t\right)=D_{g}+\int_{-\infty }^{\infty }L\left(\tau \right)\left(1-e^{-t/\tau }\right)d\ln\tau$$

where *τ* is the retardation time; *D*(*t*) is the creep compliance.

The continuous relaxation spectrum *H*(*ρ*) and continuous retardation spectrum *L*(*τ*) essentially contain identical time- and frequency-dependent material information; thus, they are equivalent of characterizing the LVE behavior of a material. As can be seen from Equations (17)–(22), once the continuous relaxation and retardation spectra are determined, both modulus and compliance functions in the time and frequency domain can be attained. In accordance with the LVE theory, the continuous spectra have the following relationships with the complex modulus E* [31]:

(23) Hρ=±π−1ImE∗iωiω→ρ−1e±iπ=±π−1ImE∗ρ−1e±iπ

(24) Lτ=∓π−1ImD∗iωiω→τ−1e±iπ=∓π−1ImD∗τ−1e±iπ=∓π−1ImE∗τ−1e±iπ−1

where Im represents the operation of retaining the imaginary part of a complex-valued function.

For the HN model, Havriliak and Negami [30] presented the analytical expression of *H*(*ρ*) through Equation (23), as follows:

(25) $$H\left(\rho \right)=\frac{\left(E_{g}-E_{e}\right){\left(\rho /\tau_{0}\right)}^{\alpha \beta }\sin\left(\beta \phi \right)}{\pi {\left[1+{\left(\rho /\tau_{0}\right)}^{2\alpha }+2{\left(\rho /\tau_{0}\right)}^{\alpha }\cos\left(\alpha \pi \right)\right]}^{\beta /2}}$$

(26) $$\phi =\arctan\frac{\sin(\alpha \pi )}{{\left(\rho /\tau_{0}\right)}^{\alpha }+\cos\left(\alpha \pi \right)}$$

Analogously, Sun et al. [26] derived the close-form solution for *L*(*τ*) of the HN model through Equation (24), as follows:

(27) $$L\left(\tau \right)=\frac{\Omega \left(E_{g}-E_{e}\right)\sin(\beta \phi )}{\pi \left\{{\left[\left(E_{e}-E_{g}\right)\cos\left(\beta \phi \right)+E_{g}\Omega \right]}^{2}+{\left[\left(E_{e}-E_{g}\right)\sin\left(\beta \phi \right)\right]}^{2}\right\}}$$

(28) $$\Omega ={\left[1+{\left(\tau_{0}/\tau \right)}^{2\alpha }+2{\left(\tau_{0}/\tau \right)}^{\alpha }\cos\left(\alpha \pi \right)\right]}^{\beta /2}$$

For the 2S2P1D model, Alavi et al. [32] obtained the analytical representation of *H*(*ρ*) through Equation (23), as follows:

(29) $$H\left(\rho \right)=\frac{\left(E_{g}-E_{e}\right)X}{\pi \left(X^{2}+Y^{2}\right)}$$

(30) $$X=1+\alpha \tau_{0}^{-k}\rho^{k}\cos\left(k\pi \right)+\tau_{0}^{-h}\rho^{h}\cos\left(h\pi \right)-\beta^{-1}\tau_{0}^{-1}\rho$$

(31) $$Y=\alpha \tau_{0}^{-k}\rho^{k}\sin\left(k\pi \right)+\tau_{0}^{-h}\rho^{h}\sin\left(h\pi \right)$$

Sun et al. [33] successfully deduced the analytical expression for *L*(*τ*) of the 2S2P1D, as follows:

(32) $$L(\tau )=\frac{Y(E_{g}-E_{e})(X^{2}+Y^{2})}{\pi \left\{{\left[E_{e}(X^{2}+Y^{2})+X(E_{g}-E_{e})\right]}^{2}+{\left[Y(E_{g}-E_{e})\right]}^{2}\right\}}$$

It is noted that *ρ* in *X* and *Y* of Equation (32) should be replaced by *τ*.

Evidently, for the HN and 2S2P1D models, the corresponding relaxation modulus *E*(*t*) and creep compliance *D*(*t*) in the time domain can be readily calculated with the continuous relaxation and retardation spectra according to Equations (19) and (22). Further, in terms of the Boltzmann superposition integrals, the constitutive relationships for the LVE material can be determined [31].

### 3.3. Construction of Master Curves

Asphalt concrete is a typical thermorheologically simple material in the LVE region; therefore, the master curves for various LVE material functions in both frequency and time domains can be constructed in accordance with the time–temperature superposition principle (TTSP). During this process, viscoelastic test data measured at different temperatures is shifted horizontally along the frequency or time axis on the logarithmic scale, thus generating a smooth master curve at a given reference temperature *T*_r_. By means of the constructed master curve, the LVE behavior over a wider range of loading time or frequency than that offered by the test instrument can be predicted. The reduced angular frequency *ω*_r_ and reduced time *t*_r_ for the shifted test data are represented by:

(33) $$\omega_{r}=\omega \times \alpha_{T}$$

(34) $$t_{r}=\frac{t}{\alpha_{T}}$$

where *α_T_* is the time-temperature shift factor. The time–temperature shift factors can be represented using a function of temperature, e.g., the Williams–Landel–Ferry (WLF) or the Arrhenius equation, or in a non-functional form. To avoid the effect of the functional expression of *α_T_*, the non-functional method was adopted for constructing the master curve of the complex modulus in the present study.

The parameters of the complex modulus model and time-temperature shift factors were determined simultaneously through a nonlinear optimization process. To fully extract the LVE information, both dynamic modulus and phase angle test data were taken into account, and the target error function to minimize was as the following:

(35) $$F=\frac{1}{N}\sqrt{{\sum_{i=1}^{N}\left(\frac{\left|E_{m,i}^{*}\right|-\left|E_{c,i}^{*}\right|}{\left|E_{m,i}^{*}\right|}\right)}^{2}}+\frac{1}{N}\sqrt{{\sum_{i=1}^{N}\left(\frac{\varphi_{m,i}-\varphi_{c,i}}{\varphi_{m,i}}\right)}^{2}}$$

where *N* is the number of the dynamic modulus or phase angle data points; $\left|E_{m,i}^{*}\right|$ and $\varphi_{m,i}$ are the measured values for the dynamic modulus and phase angle, respectively; $\left|E_{c,i}^{*}\right|$ and $\varphi_{c,i}$ are the calculated values for the dynamic modulus and phase angle from the master curve model used, respectively. The optimization operation can be easily completed using the Solver in Microsoft Excel. Before this, initial values for both master curve model parameters and shift factors should be given. The reference temperatures for Mix-13.2 and Mix-9.5 were set to 20 and 24 °C, respectively.

### 3.4. Determination of Prony Series Parameters

As stated above, once the parameters of the complex-valued models, like HN and 2S2P1D models, are known, the corresponding *H*(*ρ*) and *L*(*τ*) can be automatically determined due to the existence of their analytical expressions with the same parameters as the original complex modulus models. Although all the modulus and compliance functions can further be straightforward calculated with *H*(*ρ*) and *L*(*τ*) through Equations (17)–(22), the integral forms based on the continuous spectra are actually inconvenient to implement in numerical simulation techniques, e.g., the finite element method. Instead, the Prony series expressions on the basis of discrete spectra have been extensively utilized due to their advantage at computation efficiency.

The relaxation modulus expression derived from the generalized Maxwell model and the creep compliance expression derived from the generalized Kelvin model are two typical Prony series representations. For the generalized Maxwell model, the modulus functions can be formulated by [31]:

(36) $$E\left(t\right)=E_{e}+\sum_{j=1}^{n}E_{j}e^{-t/\rho_{j}}$$

(37) $$E^{\prime }\left(\omega \right)=E_{e}+\sum_{j=1}^{n}E_{j}\frac{\omega^{2}\rho_{j}^{2}}{1+\omega^{2}\rho_{j}^{2}}$$

(38) $$E^{\prime \prime }\left(\omega \right)=\sum_{j=1}^{n}E_{j}\frac{\omega \rho_{j}}{1+\omega^{2}\rho_{j}^{2}}$$

where *E_j_* is the modulus of the spring or the relaxation strength; *ρ_j_* = *η_j_*/*E_j_* is the discrete relaxation time; *η_j_* is the viscosity of the dashpot; the set of Prony series parameters [*ρ_j_*, *E_j_*] is called the discrete relaxation spectrum.

For the generalized Kelvin model, the compliance functions can be represented by [31]:

(39) $$D\left(t\right)=D_{g}+\sum_{j=1}^{n}D_{j}\left(1-e^{-t/\tau_{j}}\right)$$

(40) $$D^{\prime }(\omega )=D_{g}+\sum_{j=1}^{n}D_{j}\frac{1}{1+\omega^{2}\tau_{j}^{2}}$$

(41) $$D^{\prime \prime }(\omega )=\sum_{j=1}^{n}D_{j}\frac{\omega \tau_{j}}{1+\omega^{2}\tau_{j}^{2}}$$

where *D_j_* is the compliance of the spring or the retardation strength; *τ_j_* = *λ_j_D_j_* is the discrete retardation time; *λ_j_* is the viscosity of the dashpot; the set of Prony series parameters [*τ_j_*, *D_j_*] is called the discrete retardation spectrum.

In fact, when the discrete relaxation and retardation spectra become infinitely dense, they evolve into the so-called continuous relaxation and retardation spectra. As such, Equations (36)–(41) can be interpreted as discretizations of Equations (17)–(22). For the storage modulus *E*′(*ω*), *H*(*ρ*)*d*ln*ρ* in Equation (17) represents the contribution of the model to the modulus function in the interval of ln*ρ* and ln*ρ*+*d*ln*ρ*, which leads to the following derivation:

(42) $$\begin{array}{cc} E^{\prime }\left(\omega \right) & =E_{e}+\int_{-\infty }^{\infty }H\left(\rho \right)\frac{\omega^{2}\rho^{2}}{1+\omega^{2}\rho^{2}}d\ln\rho \\ & \approx E_{e}+\sum_{j=1}^{n}\left[H\left(\rho_{j}\right)\times \Delta \ln\rho_{j}\right]\frac{\omega^{2}\rho_{j}^{2}}{1+\omega^{2}\rho_{j}^{2}}\\ & =E_{e}+\sum_{j=1}^{n}E_{j}\frac{\omega^{2}\rho_{j}^{2}}{1+\omega^{2}\rho_{j}^{2}} \end{array}$$

Likewise, for the storage compliance *D*′(*ω*), the integral form based on the continuous retardation spectrum and the series expression based on the discrete retardation spectrum have the following relationship:

(43) $$\begin{array}{cc} D^{\prime }\left(\omega \right) & =D_{g}+\int_{-\infty }^{\infty }L\left(\tau \right)\frac{1}{1+\omega^{2}\tau^{2}}d\ln\tau \\ & \approx D_{g}+\sum_{j=1}^{n}\left[L\left(\tau_{j}\right)\times \Delta \ln\tau_{j}\right]\frac{1}{1+\omega^{2}\tau_{j}^{2}}\\ & =D_{g}+\sum_{j=1}^{n}D_{j}\frac{1}{1+\omega^{2}\tau_{j}^{2}} \end{array}$$

During the determination of the Prony series parameters, the discrete time constants, *ρ_j_* and *τ_j_*, are commonly preselected. Specifically, they are set to values with equal intervals on the logarithmic scale according to Equation (44):

(44) $$\rho_{i}=\tau_{i}=b\times 10^{d+i/M}$$

where *b* and *d* are specified according to the logarithmic time range covered by the shifted test data, and generally *b* = 1; *M* is the number of the discrete times assumed in each decade on the logarithmic scale.

It can be observed that with the discrete time constants (*ρ_j_* and *τ_j_*) known, the Prony series coefficients, namely the relaxation and retardation strengths (*E_j_* and *D_j_*), can be quickly and easily calculated using the following equations:

(45) $$E_{i}=H\left(\rho_{i}\right)\times \Delta \ln\rho_{i}$$

(46) $$D_{i}=L\left(\tau_{i}\right)\times \Delta \ln\tau_{i}$$

(47) $$\Delta \ln\rho_{i}=\Delta \ln\tau_{i}=\frac{1}{M}\ln10$$

Finally, the remaining two elastic constants *E*_e_ and *D*_g_ can be determined by fitting Equations (37) and (40) to the corresponding real part expression of the original complex modulus model, like Equation (6) or (10), over the range covered by the shifted test data through the Excel Solver. In such a manner, all the Prony series parameters can be fast acquired. Actually, *E*_e_ and *D*_g_ have an analytical relationship, as follows:

(48) $$D_{g}=\frac{1}{E_{g}}=\frac{1}{E_{e}+\sum_{j=1}^{n}E_{j}}$$

Therefore, once the elastic constant *E*_e_ along with the discrete relaxation strengths *E_j_* is available, *D*_g_ can be obtained accordingly.

## 4. Results and Discussion

### 4.1. Examination of Test Data Quality of Asphalt Concrete

Before constructing the master curves, it is crucial to examine the quality of the complex modulus measurements. In the present study, the Black diagram [34] was employed to conduct this manipulation, in which the dynamic modulus |E*| is plotted against the phase angle *φ* in a single plane. Since for a thermorheologically simple material, all the components of the complex modulus are the functions of the reduced angular frequency, any two of them can form a unique curve in a complex plane. In the Black diagram, the angular frequency axis can be treated as an additional axis perpendicular to the complex plane in accordance with the right-hand rule. Thus, the testing temperatures would have no effect on the analysis of the overlapping behavior of the test data during the construction of master curves in the Black diagram. A smoother Black curve generally represents a higher quality of the test data. In such a manner, the Black diagram allows an effective and efficient detection of inconsistency with thermorheological simplicity.

**Figure 2:**
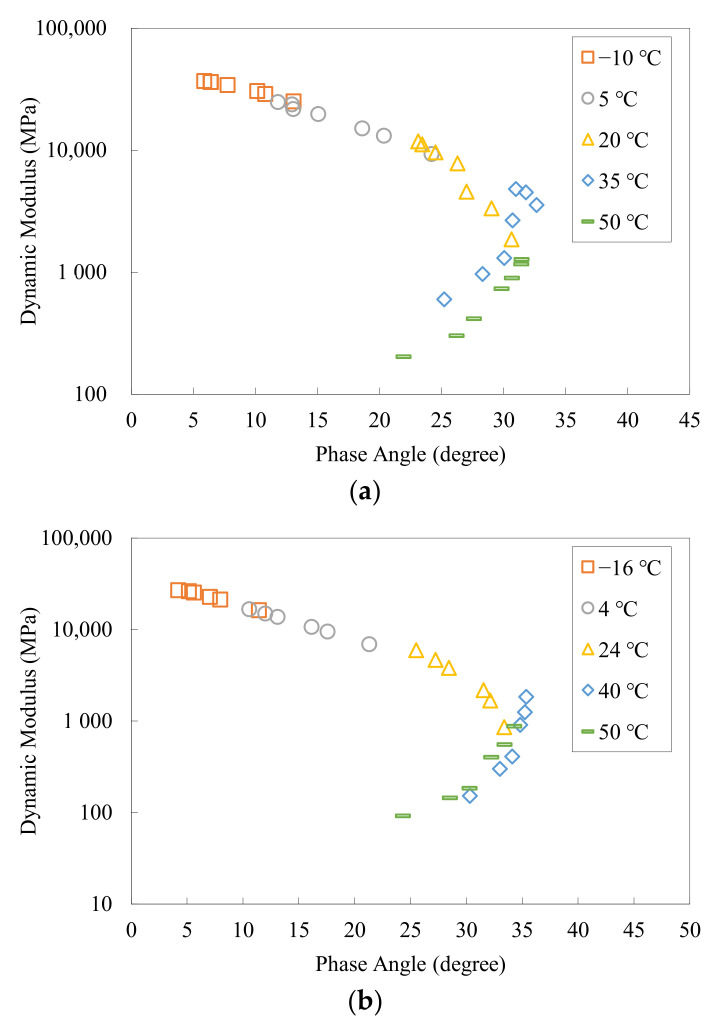
Black diagram of the complex modulus test data set: (**a**) Mix-13.2; (**b**) Mix-9.5.

Figure 2 shows the resulting Black diagrams for the two asphalt mixtures. It can be observed that in both diagrams, the complex modulus test data obtained at different temperatures basically formed unique curves, indicating the compliance with thermorheological simplicity under the test conditions as well as the applicability of the TTSP. In addition, the test results at lower temperatures exhibited better overlapping behavior, whereas those at higher temperatures showed slightly higher dispersion. This is mainly because nonlinear behaviors (e.g., the viscoplastic deformation) of asphalt concrete occur more easily at higher temperatures, which impact the measurement of LVE responses of the material to a certain degree.

### 4.2. Analysis of Results from the Developed Method

**Figure 3:**
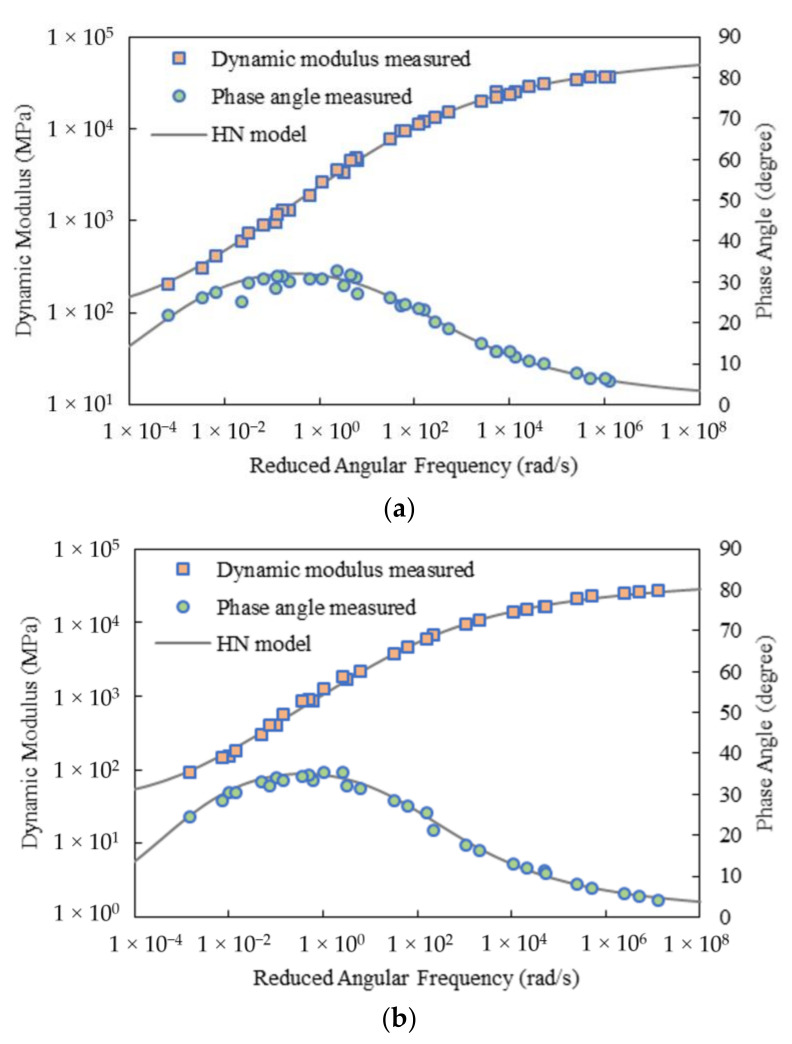
Master curves of dynamic modulus and phase angle from the HN model: (**a**) Mix-13.2; (**b**) Mix-9.5.

**Figure 4:**
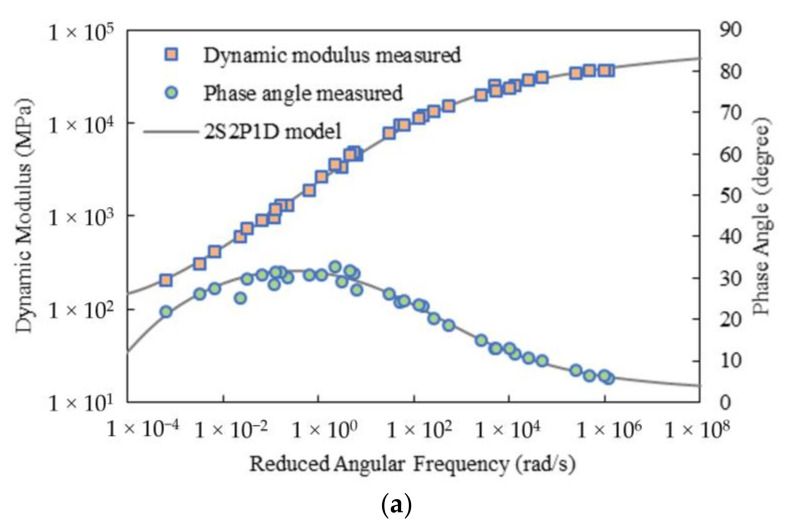

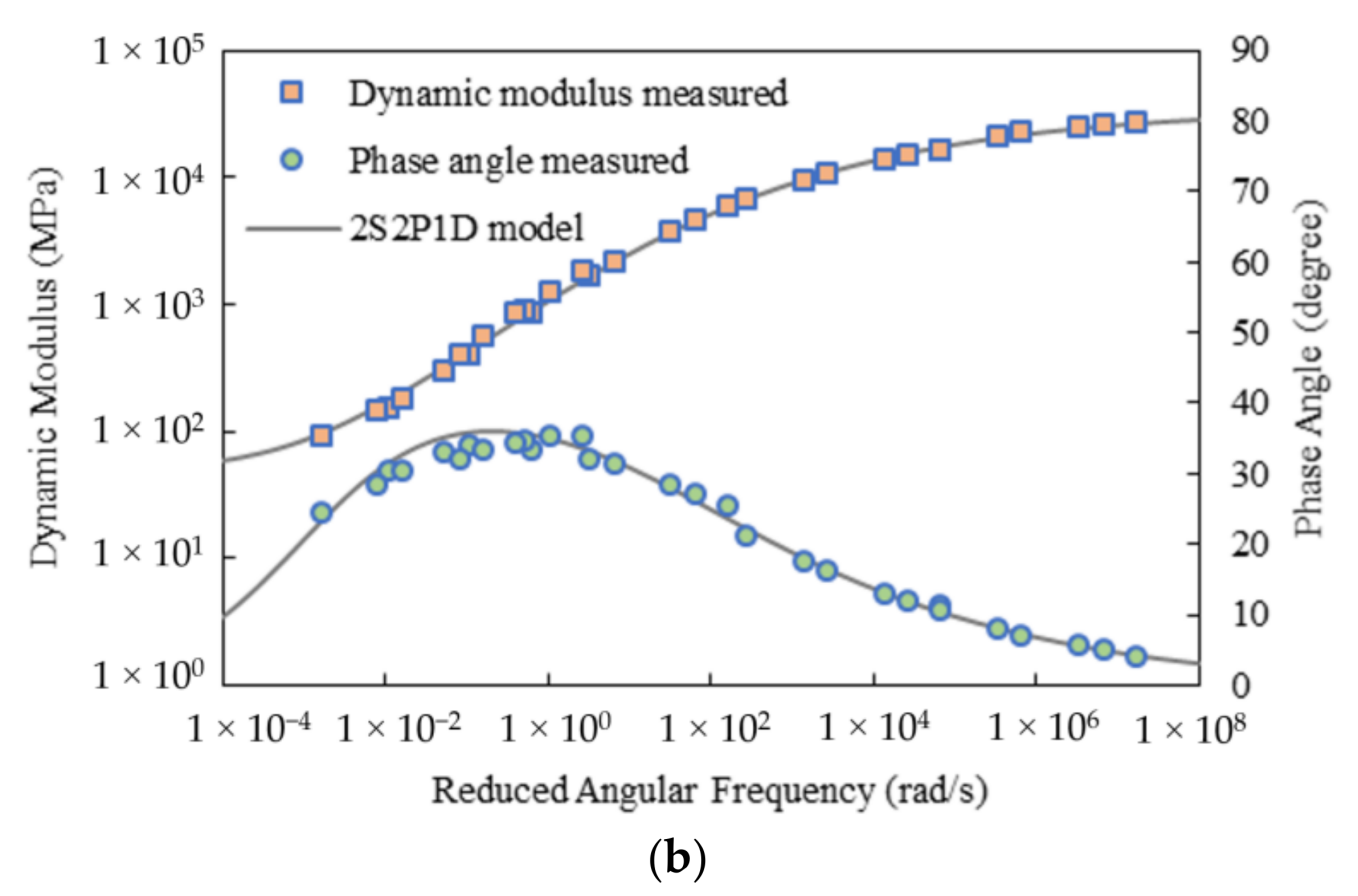
Master curves of dynamic modulus and phase angle from the 2S2P1D model: (**a**) Mix-13.2; (**b**) Mix-9.5.

**Figure 5:**
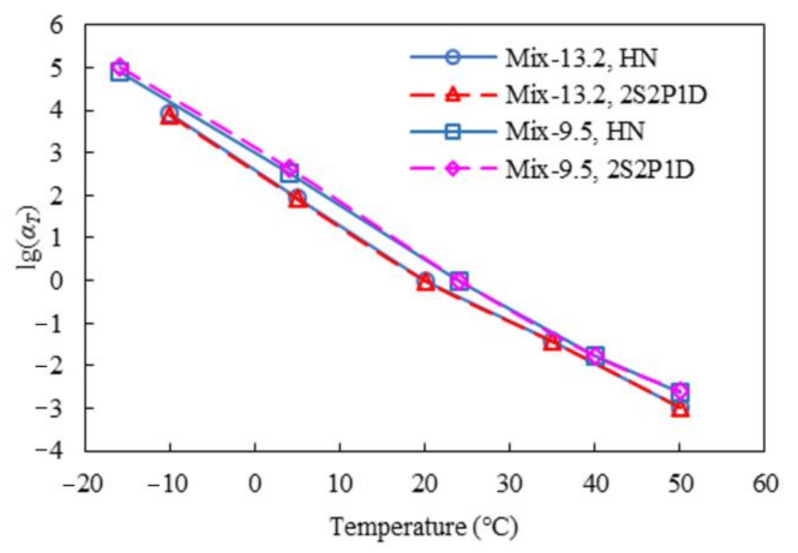
Time-temperature shift factors obtained using the HN and 2S2P1D methods.

Figure 3 and Figure 4 present the master curves of the dynamic modulus and phase angle respectively developed from the HN and 2S2P1D models. As observed, both models fitted to the test data of the two mixtures very well. Table 1 and Table 2 list the resulting model parameters and fitting errors. For Mix-13.2, the two complex modulus models contributed to very close fitting errors, whereas for Mix-9.5, the 2S2P1D model yielded slightly lower fitting error than that from the HN model. This may be because the 2S2P1D model has more parameters and thus higher flexibility. Besides, the time-temperature shift factors calculated using the HN and 2S2P1D methods were found very close as well, as shown in Figure 5.

**Figure 6:**
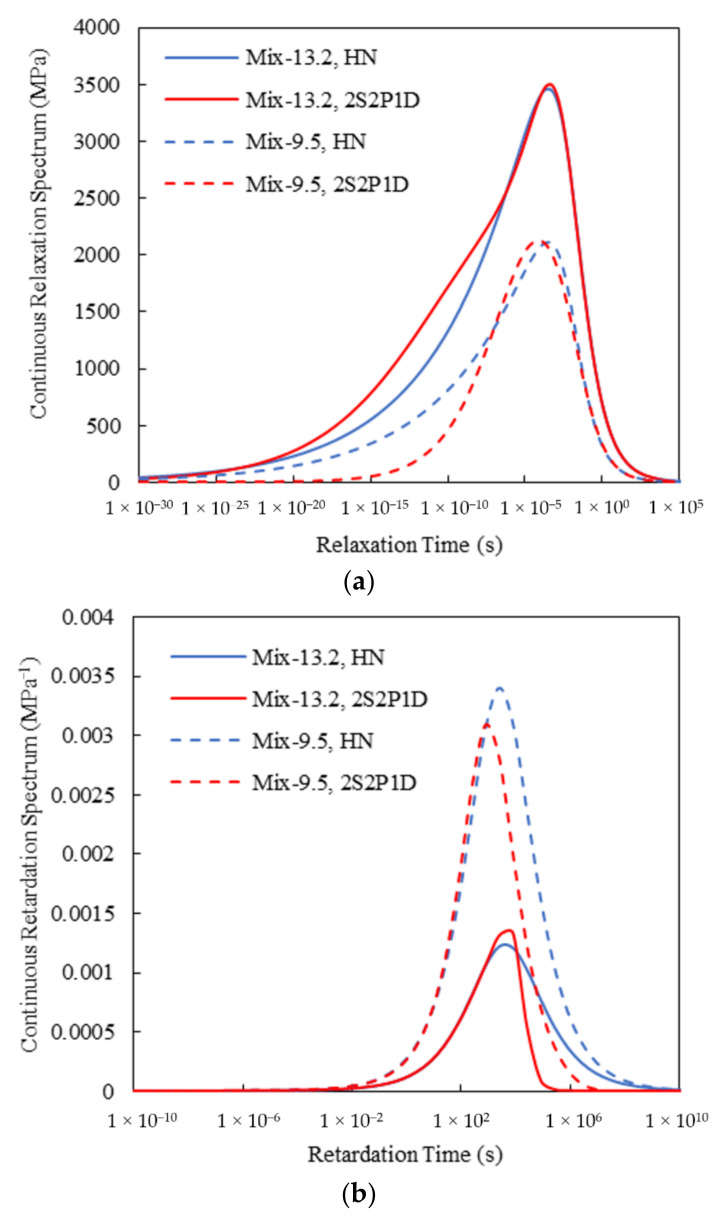
Continuous spectra of the two asphalt mixtures: (**a**) Relaxation; (**b**) Retardation.

As mentioned previously, with the obtained complex modulus model parameters, both continuous relaxation and retardation spectra can be analytically developed [see Equations (25)–(32)]. Figure 6 shows the continuous spectra of the two asphalt mixtures. It can be observed that for both mixtures, the HN and 2S2P1D models exhibited slightly different continuous spectral patterns, particularly at shorter relaxation times and longer retardation times. However, at the time range of 10^−8^ to 10^4^ s, which is approximately corresponding to the angular frequency range of 10^−4^ to 10^8^ rad/s, that is, the region mostly covered by the test data (Figure 3 and Figure 4), the continuous spectra for the HN and 2S2P1D models were very close to each other.

Based on the continuous spectra developed, the corresponding discrete spectra can be fast determined using Equations (44)–(47). Although the relaxation and retardation times of the discrete spectra can be preset at any time regions of interest with any widths, it is a common practice that they are selected at regions covered by test data [3,12,35]. In this way, the numbers of the Prony series parameters can be reduced reasonably without losing significant computation accuracy. Consequently, the range of the discrete spectra was selected at 10^−8^ to 10^4^ s in this study.

**Figure 7:**
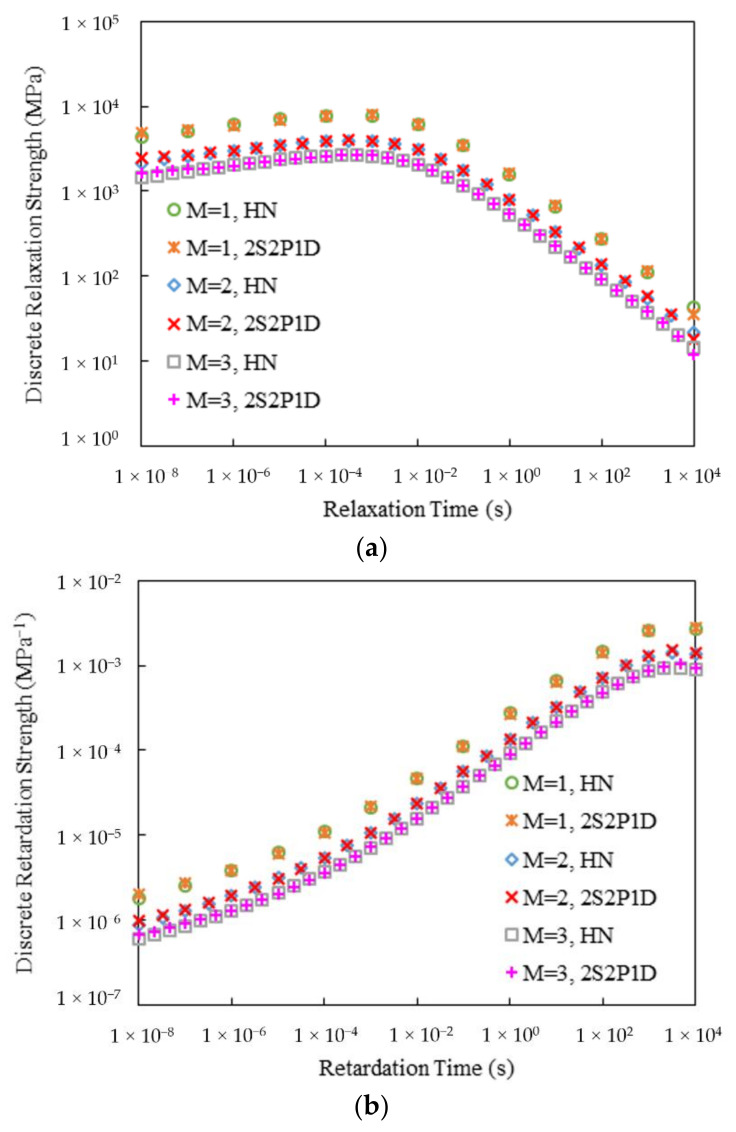
Discrete spectra of Mix-13.2: (**a**) Relaxation; (**b**) Retardation

**Figure 8:**
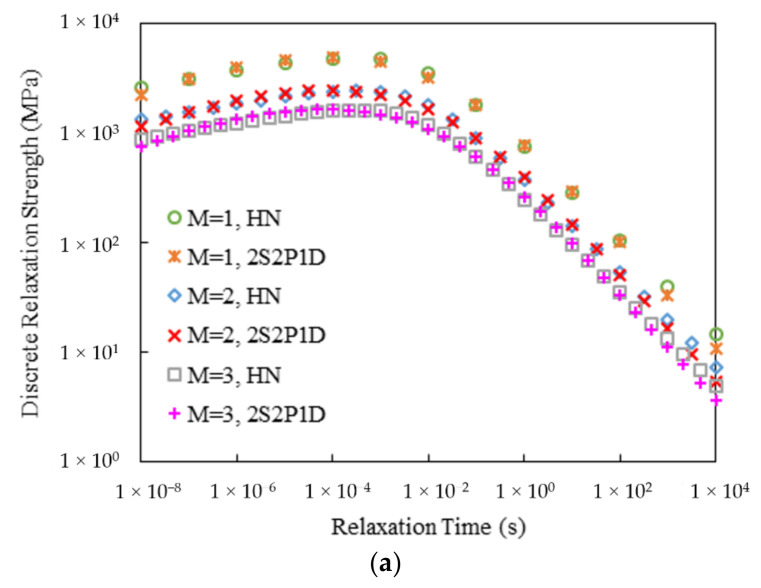

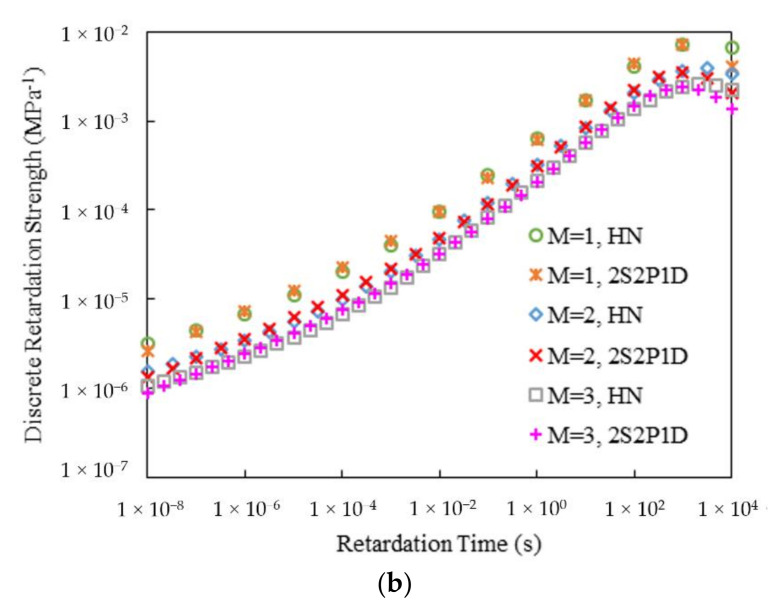
Discrete spectra of Mix-9.5: (**a**) Relaxation; (**b**) Retardation.

Figure 7 and Figure 8 display the calculated discrete relaxation and retardation spectra for the two mixtures. Three densities of the discrete spectrum lines, namely, *M* = 1, 2 and 3, were considered. As can be seen, the resulting discrete spectra from both HN and 2S2P1D models were very smooth without any local oscillations. Also, since the discrete spectrum strengths were all calculated from the corresponding positive continuous spectra, no negative strength values were produced.

To establish the Prony series representations for the relaxation modulus *E*(*t*) and creep compliance *D*(*t*) in Equations (36) and (39), the elastic constants *E*_e_ and *D*_g_ need to be further determined. To this end, Equation (37) for the storage modulus *E*′ was fitted to Equations (6) and (10) separately for the real parts of the HN and 2S2P1D models. Before fitting, smoothed data points were generated from Equations (6) and (10), equally spaced on the logarithmic scale within the region covered by the test data. With *E*_e_ determined, *D*_g_ can be fast obtained by Equation (48).

**Figure 9:**
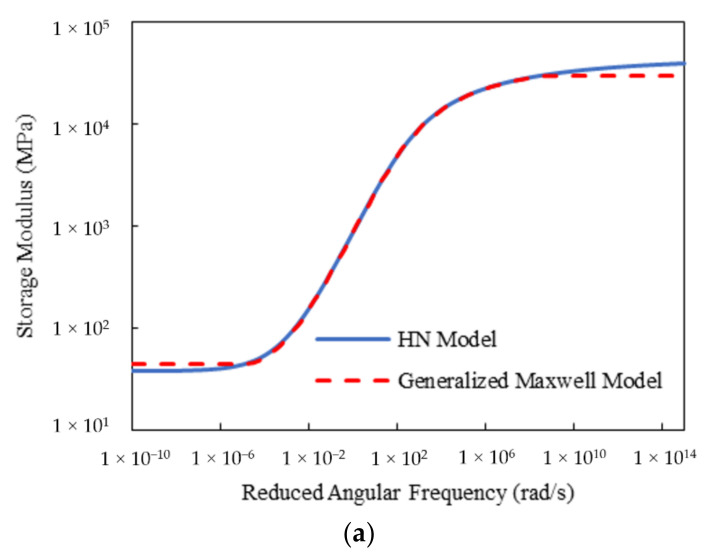

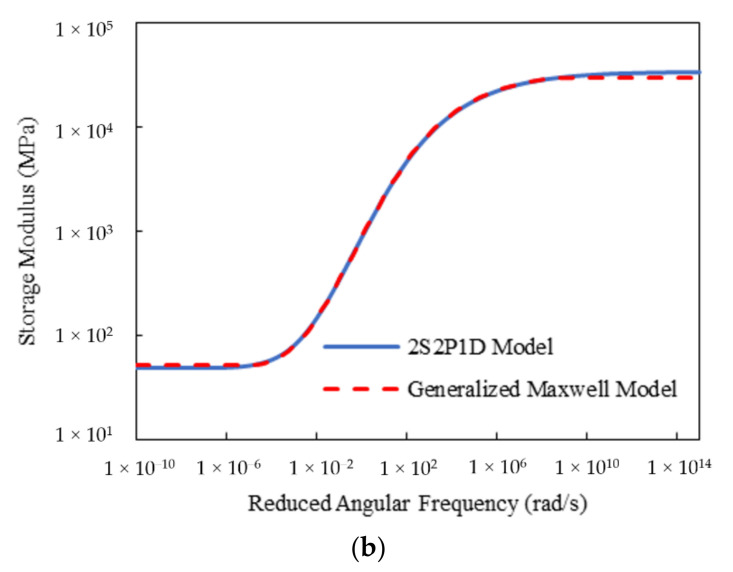
Master curves of storage modulus for Mix-9.5 using the discrete relaxation spectrum with *M* = 1 from: (**a**) HN model; (**b**) 2S2P1D model.

Figure 9 gives the developed master curves of the storage modulus for Mix-9.5 using the discrete relaxation spectrum, i.e., using the generalized Maxwell model, with *M* = 1 from the HN and 2S2P1D models, respectively. Obviously, both methods yielded satisfactory results over the region where the spectrum lines were selected. Similar observations were made for Mix-13.2. It should be mentioned that, traditionally, one spectrum line per decade (*M* = 1) is extensively accepted for generating the Prony series representation. The higher density of the spectrum lines would generate higher accuracy for fitting but would produce more Prony series parameters. Thus, in the following sections, only the results for *M* = 1 are presented.

**Figure 10:**
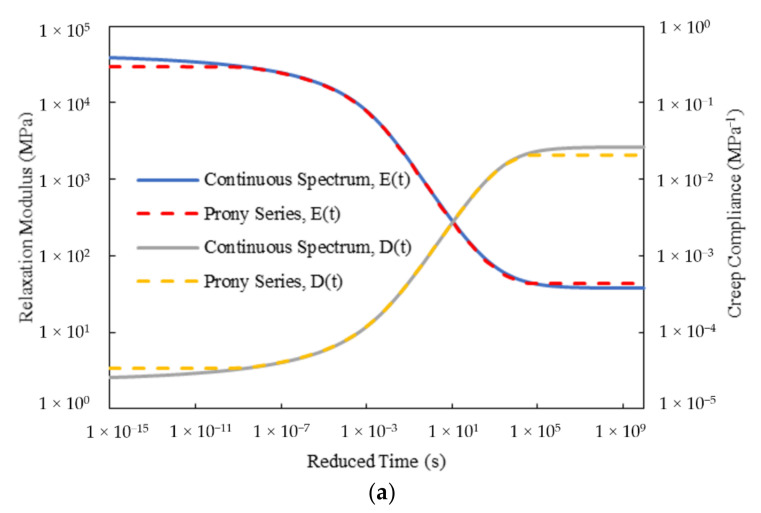

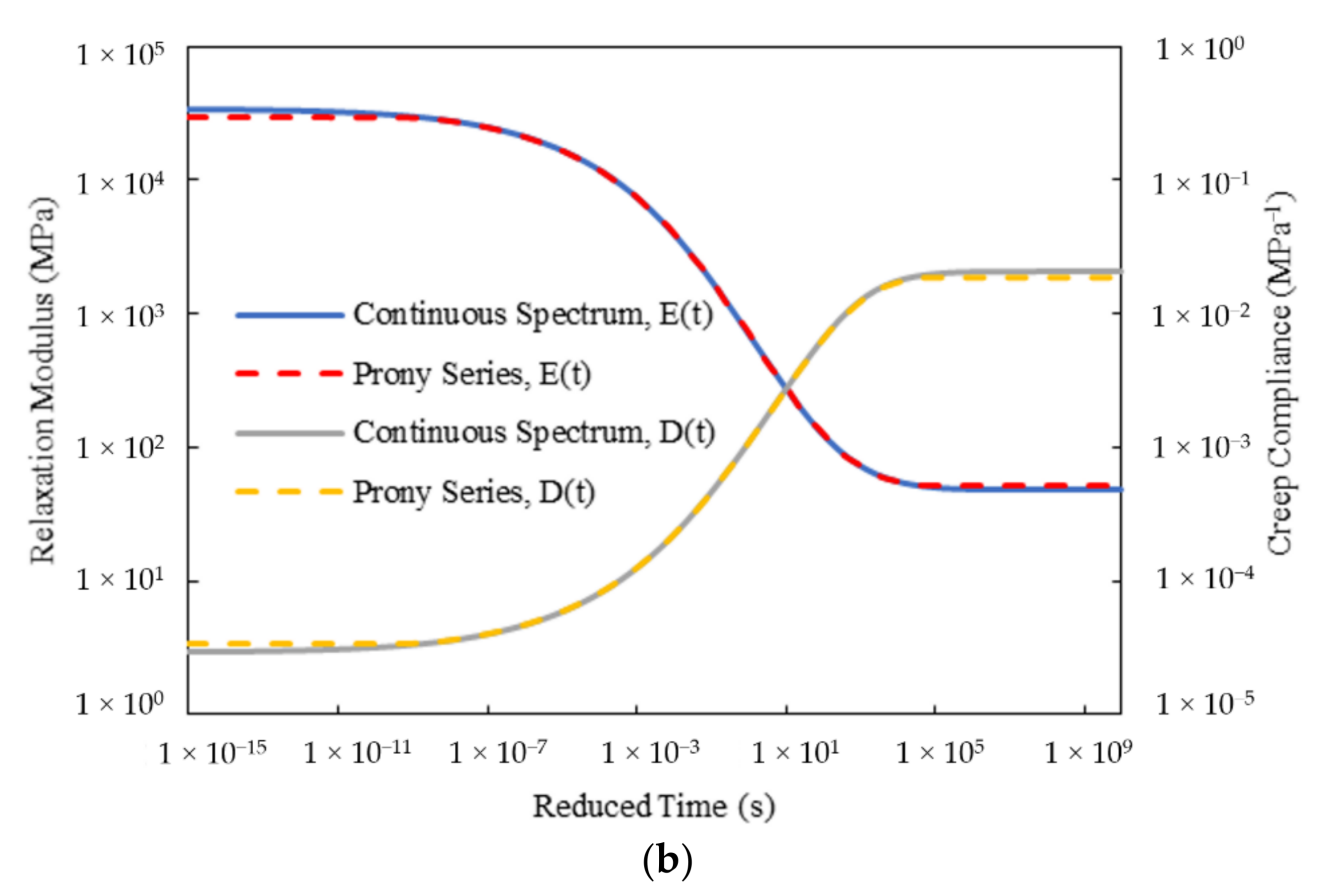
Master curves of relaxation modulus and creep compliance for Mix-9.5 in the Prony series forms from: (**a**) HN model; (**b**) 2S2P1D model.

**Figure 11:**
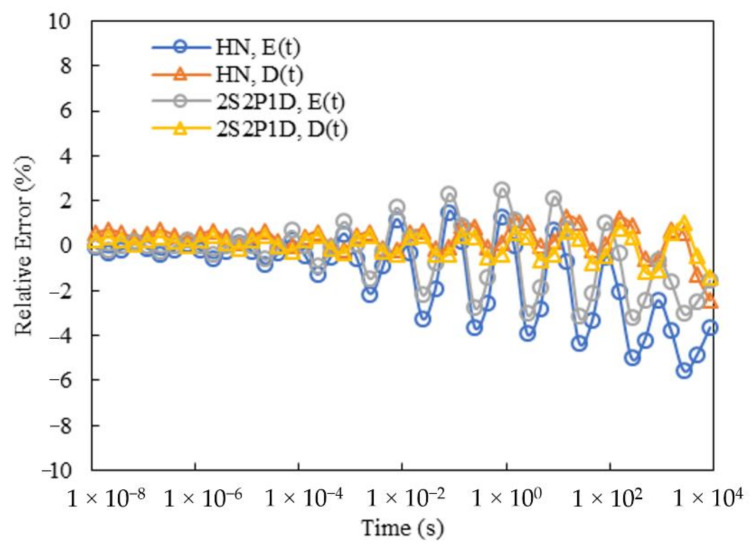
Relative errors between the master curves from Prony series forms and continuous spectra for Mix-9.5.

Figure 10 gives the master curves of the relaxation modulus and creep compliance for Mix-9.5 in the Prony series forms from the HN and 2S2P1D models. To verify the quality of the calculated Prony series parameters, the corresponding curves developed through the continuous spectra are also presented. Figure 11 gives the relative errors between the master curves from the Prony series forms and continuous spectra for Mix-9.5. It should be mentioned that since the spectrum lines were selected only at the time range covered by the test data, only the relative errors at 10^−8^ to 10^4^ s were calculated. To achieve the infinite integrals in Equations (19) and (22), an integral interval of 10^−40^ to 10^+40^ s was employed to approximately represent the infinite one through the trapezoidal rule, in which 100 increments per decade were equidistantly selected on the logarithmic time scale. It can be seen that the curves from the Prony series parameters were in good agreement with those from the continuous spectra for both HN and 2S2P1D models over the region where the spectrum lines were selected, thus demonstrating the effectiveness of the proposed method in this study. Equally desirable results were also found for Mix-13.2.

### 4.3. Comparison to the Conventional Sigmoidal Model Method

Considering that the quality of the resulting Prony series parameters are dependent on the master curve models used for presmoothing, the results obtained were compared with those from the Sigmoidal model, which has been adopted by MEPDG [36]. The Sigmoidal model with four parameters can be expressed by:

(49) $$\mathrm{lg}\left(\left|E^{*}\right|\right)=a_{1}+\frac{a_{2}}{1+e^{a_{3}+a_{4}\mathrm{lg}\omega }}$$

where *a*_1_ is the on the minimum logarithmic value of the dynamic modulus; *a*_2_ is the difference of the maximum and minimum logarithmic values of the dynamic modulus; *a*_3_ and *a*_4_ are model parameters governing the curve shape.

Unlike the HN and 2S2P1D models, the Sigmoidal function is a real-valued model for the dynamic modulus, and thus does not have an accurate analytical model for the corresponding phase angle. To deal with this issue, Rowe [37] developed a representation for the phase angle using an approximate Kronig–Kramers relation [38], as follows:

(50) $$\varphi \approx 90\times \frac{d\mathrm{lg}\left(\left|E^{*}\right|\right)}{d\mathrm{lg}\omega }=-90a_{2}a_{4}\frac{e^{a_{3}+a_{4}\mathrm{lg}\omega }}{{\left(1+e^{a_{3}+a_{4}\mathrm{lg}\omega }\right)}^{2}}$$

**Figure 12:**
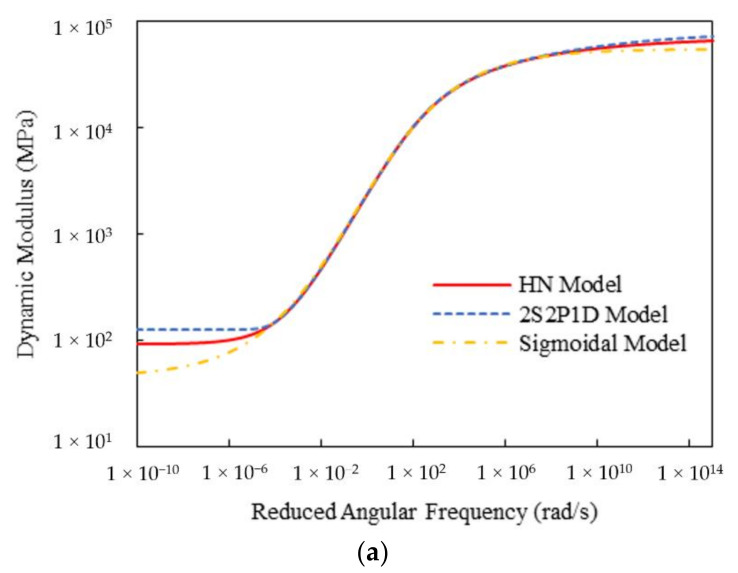

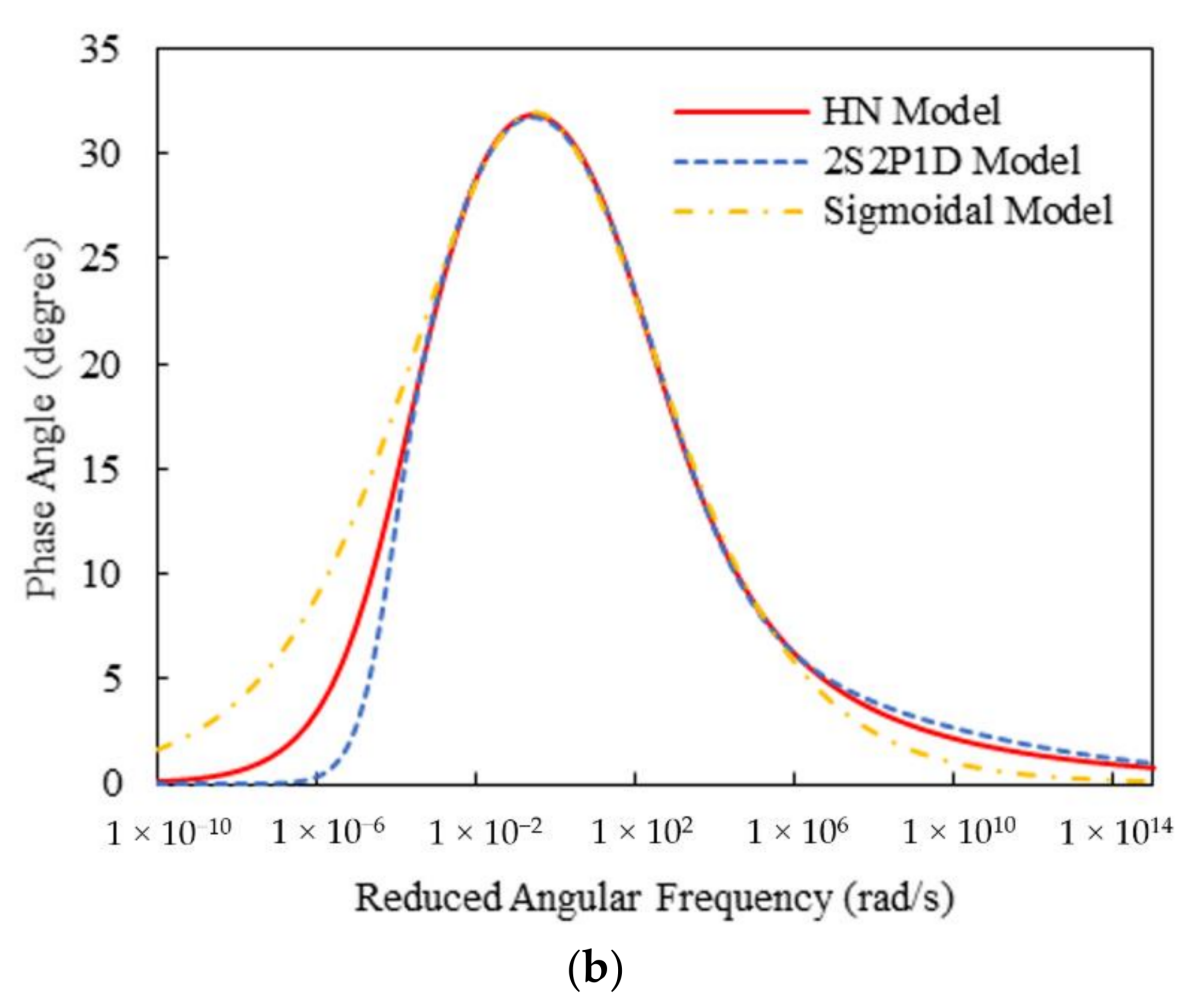
Comparison of master curves of dynamic modulus and phase angle for Mix-13.2: (**a**) dynamic modulus; (**b**) phase angle.

**Figure 13:**
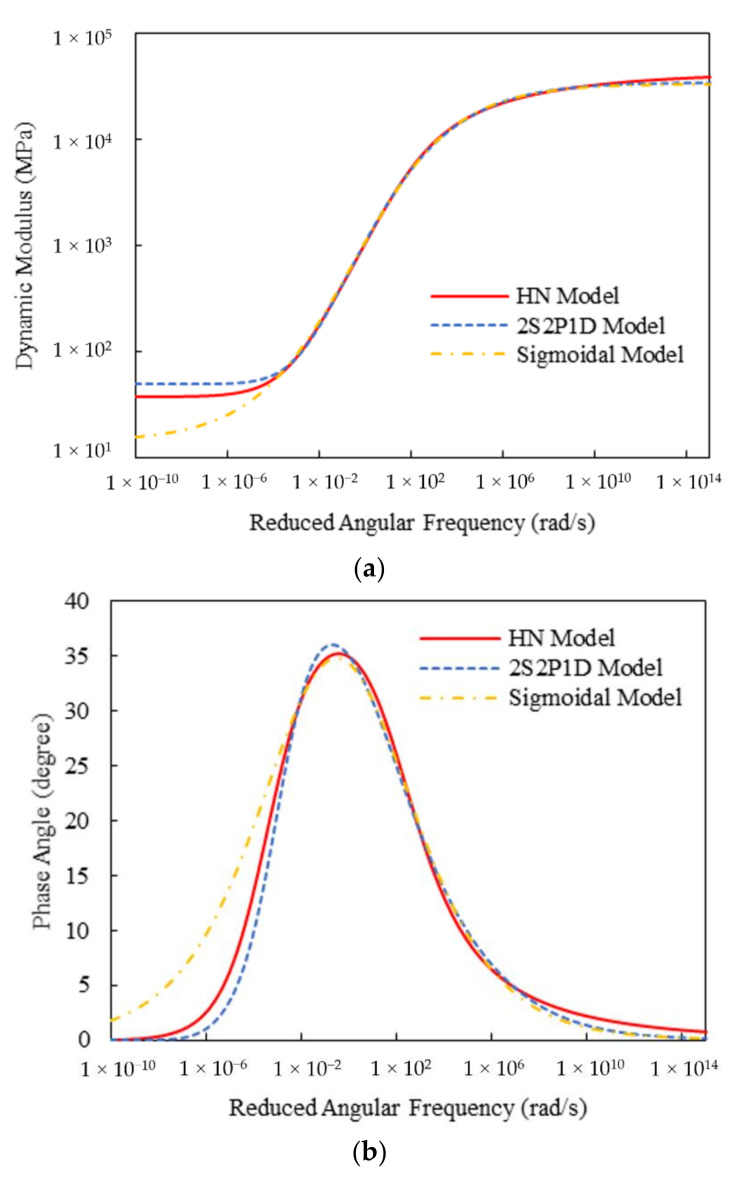
Comparison of master curves of dynamic modulus and phase angle for Mix-9.5: (**a**) dynamic modulus; (**b**) phase angle.

Table 3 shows the calculated Sigmoidal model parameters and fitting errors for the two mixtures. It can be observed that both HN and 2S2P1D models generated lower fitting errors than the Sigmoidal model, indicating their higher applicability to the complex modulus test data. To gain an in-depth insight into their advantages, the master curves of the dynamic modulus and phase angle for the three models were plotted in Figure 12 and Figure 13. It can be found that for the dynamic modulus, the curves from both HN and 2S2P1D models are non-centrosymmetric, that is, they offer asymmetric inflection points, whereas the Sigmoidal model is centrosymmetric on the log-log scale. As a result, the HN and 2S2P1D models exhibit higher flexibility than the Sigmoidal model in modeling the dynamic modulus of asphalt concrete. Additionally, the phase angle master curves of the HN and 2S2P1D models are non-axisymmetric, while that for the Sigmoidal is axisymmetric. Evidently, non-axisymmetric curves are more suitable for simulating the phase angle test data of asphalt concrete.

As a representation for the dynamic modulus, the Sigmoidal model does not have corresponding close-form solutions for *H*(*ρ*) or *L*(*τ*). Thus, the Prony series parameters for the relaxation modulus and creep compliance cannot be analytically yielded. To obtain the Prony series parameters, only the numerical approach can be used, in which the storage modulus representation from the generalized Maxwell model in Equation (37) is directly fitted to smoothed data produced from Equations (49) and (50). Similarly, the Prony series for the creep compliance also needs to be numerically computed. In this regard, the complex-valued models adopted in this study, like the HN and 2S2P1D models, have the prominent advantage over real-valued models.

**Figure 14:**
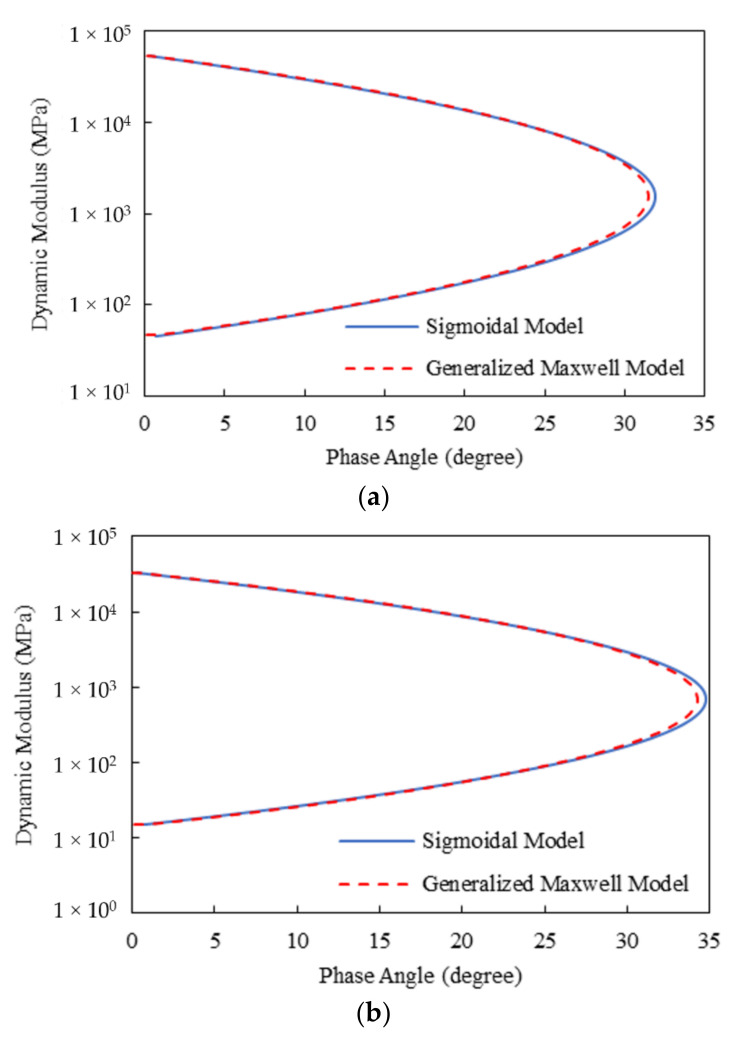
Black diagrams from the Sigmoidal model for the two asphalt mixtures: (**a**) Mix-13.2; (**b**) Mix-9.5.

Figure 14 displays the Black diagrams plotted using the Sigmoidal model for the two asphalt mixtures. For a comparison purpose, the generalized Maxwell model developed using the storage modulus data generated from the Sigmoidal model is also shown. To guarantee a good consistency of the generalized Maxwell model to the smoothed storage modulus data, the recursive fitting method developed by Sun et al. [14] was utilized. It can be clearly seen from Figure 14 that the curves from the two models diverge around the peaks of the phase angle, which indicates a noncompliance of the Sigmoidal model method with the LVE theory. This is ascribed to the use of approximate Kronig–Kramers relation. In this connection, the HN and 2S2P1D models employed in the presented approach can accurately satisfy the Kronig–Kramers relation due to the presence of the analytical representations of both the real and imaginary parts of the complex modulus.

## 5. Summary and Conclusions

This study presented a practical approach to fast acquiring high-quality Prony series parameters for both relaxation modulus and creep compliance of asphalt concrete based on the complex modulus test data. The approach can directly determine Prony series parameters through the analytical representations of the continuous relaxation and retardation spectra from the HN and 2S2P1D complex modulus models. With the model parameters determined in constructing dynamic modulus and phase angle master curves, the Prony series parameters can be immediately obtained with required accuracy. The elastic constants, *E*_e_ and *D*_g_, for both LVE modulus and compliance functions can further be readily calculated through the smoothed data from the storage modulus representations of the HN and 2S2P1D complex modulus models. To offer an in-depth interpretation for the approach, the performance of the HN, 2S2P1D and conventional Sigmoidal models in fitting the complex modulus master curves were compared. Based on the results and analysis from this study, main conclusions can be drawn as follows:

(1) The HN and 2S2P1D models yielded slightly different continuous spectral patterns at shorter relaxation times and longer retardation times. However, at the region covered by the test data, the continuous spectra of the two complex modulus models were very close to each other. Thus, the two models can generate comparable Prony series parameters within the time or frequency range covered by test data.
(2) By means of the positive analytical expressions of the continuous spectra, local spectrum oscillations and undesirable negative spectrum strengths were successfully eliminated, thus generating high-quality Prony series parameters.
(3) The HN and 2S2P1D models provide non-centrosymmetric curve patterns for the dynamic modulus master curves on the log-log scale and non-axisymmetric curve patterns for the phase angle master curves on the logarithmic angular frequency scale. Therefore, they performed better than the traditional Sigmoidal model in fitting to the complex modulus test data.
(4) The Black diagram is recommended for examining the quality of the complex modulus test data before constructing the master curves, because it can effectively avoid the effect of testing temperatures.
(5) The analytical expressions of the storage and loss moduli for both HN and 2S2P1D models accurately meet the Kronig–Kramers relation, and therefore the master curves constructed are consistent with the LVE theory.
(6) All the procedures in the proposed method can be easily achieved even only by Microsoft Excel, successfully avoiding sophisticated expertise for programming in implementation process. Thus, the proposed method furnishes a practical way to fast acquiring high-quality Prony series parameters.

Further studies are required to develop predictive models of the complex modulus master curve of asphalt concrete based on the HN and 2S2P1D models by using statistical relationships between the model parameters and the constituent and volumetric properties of asphalt concrete, and the work is ongoing.

## Figures and Tables

**Table 1:** HN model parameters and fitting errors.

Mix Type	*T*_r_/°C;	*E*_g_/MPa	*E*_e_/MPa	*α*	*β*	*τ*_0_/s	*F*/%
Mix-13.2	20	73,132	92.0	0.398	0.193	0.013	1.943
Mix-9.5	24	43,707	37.5	0.431	0.175	0.011	2.461

**Table 2:** 2S2P1D model parameters and fitting errors.

Mix Type	*T*_r_/°C;	*E*_g_/MPa	*E*_e_/MPa	*α*	*k*	*h*	*β*	*τ*_0_/s	*F*/%
Mix-13.2	20	80,329	126.2	1.805	0.104	0.412	38,400	2.485 × 10^−4^	1.961
Mix-9.5	24	34,132	49.1	2.329	0.205	0.493	41,666	1.558 × 10^−3^	2.204

**Table 3:** Sigmoidal model parameters and fitting errors.

Mix Type	*T*_r_/°C;	*a*_1_	*a*_2_	*a*_3_	*a*_4_	*F*/%
Mix-13.2	20	1.647	3.089	−0.246	−0.460	2.089
Mix-9.5	24	1.152	3.370	−0.233	−0.459	2.471

## Data Availability

Data sharing is not applicable to this article.
